# Supplementary figures and images for: Low Preoperative Antithrombin III Level Is Associated with Postoperative Acute Kidney Injury after Liver Transplantation
Source: J Pers Med. 2021 Jul 26;11(8):716. doi: 10.3390/jpm11080716 (PMC8401622; doi:10.3390/jpm11080716)

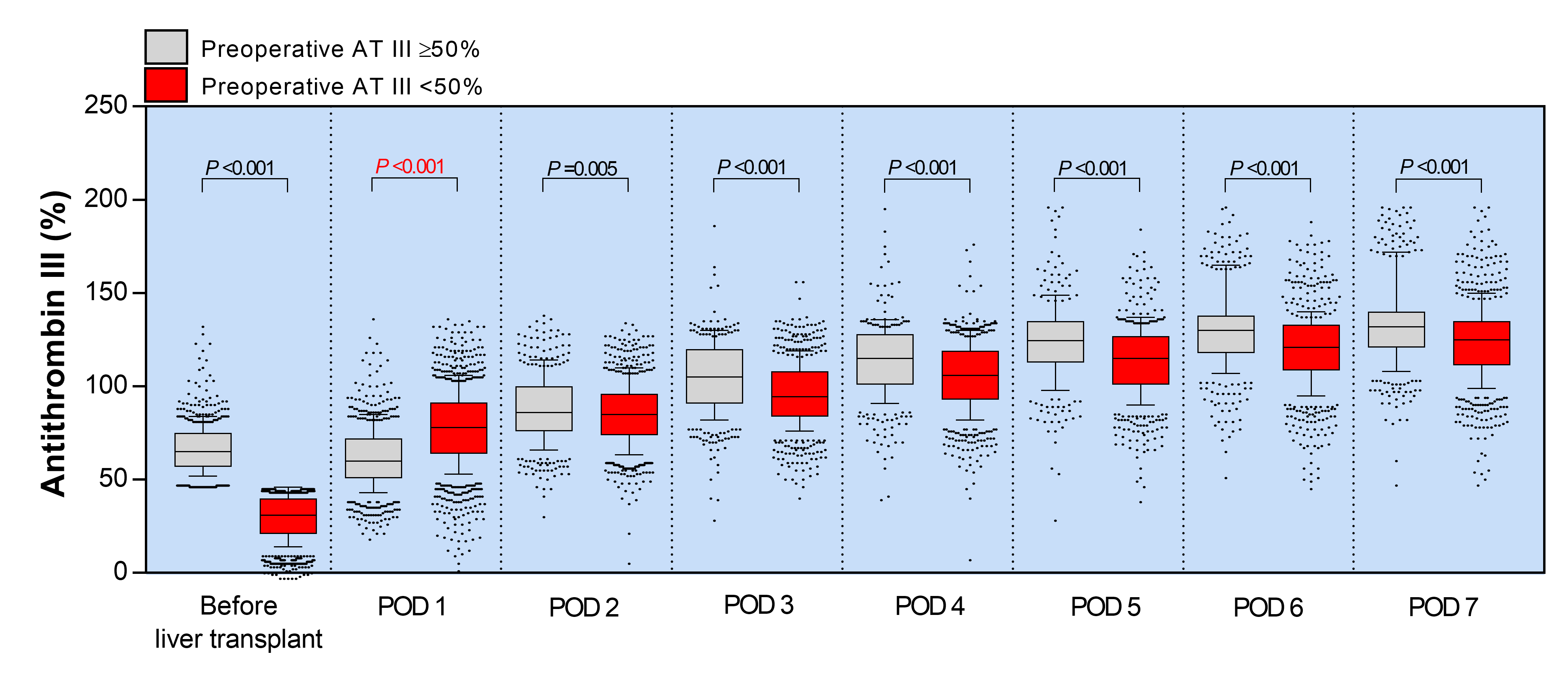

Supplement: Supplementary file 1 [file jpm-11-00716-s001.zip › suppl Fig S1.png]

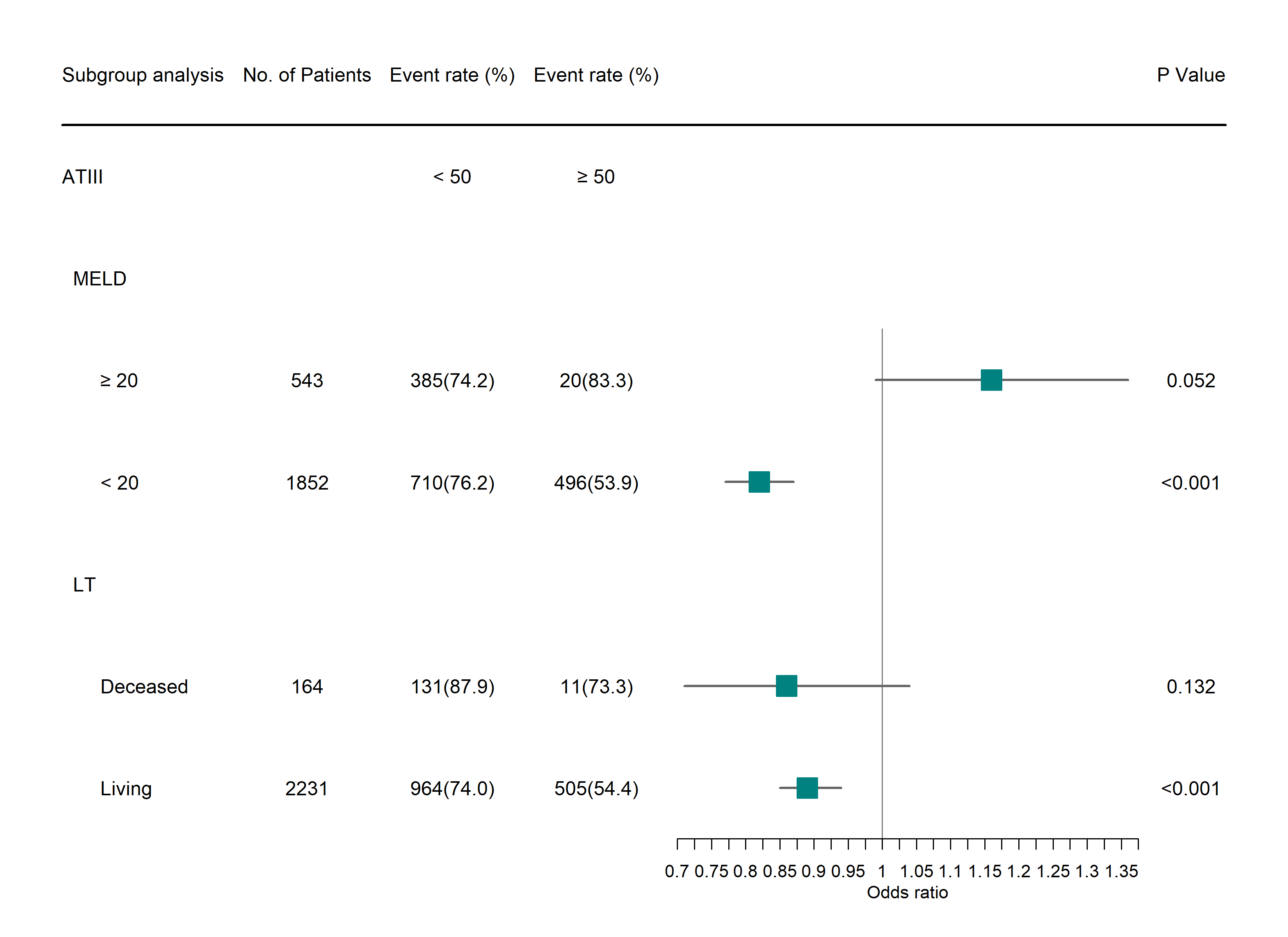

Supplement: Supplementary file 1 [file jpm-11-00716-s001.zip › suppl Fig S2.tiff]
